# Real‐World Management of Difficult Exacerbations of Bronchiectasis: A Case Study to Illustrate How Guidelines Can Support Best Practice

**DOI:** 10.1002/ccr3.73566

**Published:** 2026-09-25

**Authors:** Hulance Bianca, Capstick Toby, Shah Anand, McCallion Paul

**Affiliations:** ^1^ Department of Respiratory Medicine Torbay and South Devon NHS Foundation Trust Torquay UK; ^2^ Medicines Management and Pharmacy Services St James's University Hospital Leeds UK; ^3^ Centre for Bacterial Resistance Biology, Department of Infectious Diseases Imperial College London London UK; ^4^ Department of Respiratory Medicine Royal Brompton Hospital, Guy's and St Thomas' NHS Foundation Trust London UK; ^5^ Department of Respiratory Medicine Freeman Hospital Newcastle upon Tyne UK; ^6^ Faculty of Medical Sciences University of Newcastle Upon Tyne Newcastle upon Tyne UK

**Keywords:** bronchiectasis, case study, guidelines, respiratory, therapeutics

## Abstract

Timely management of exacerbations and optimisation of ongoing care are vital in preventing disease progression and preserving outcomes and quality of life in patients with bronchiectasis. This retrospective case review underscores the importance of the new ERS guidelines in supporting multidisciplinary teams to provide optimal care for their patients.

## 1 Introduction

**FIGURE 1:**
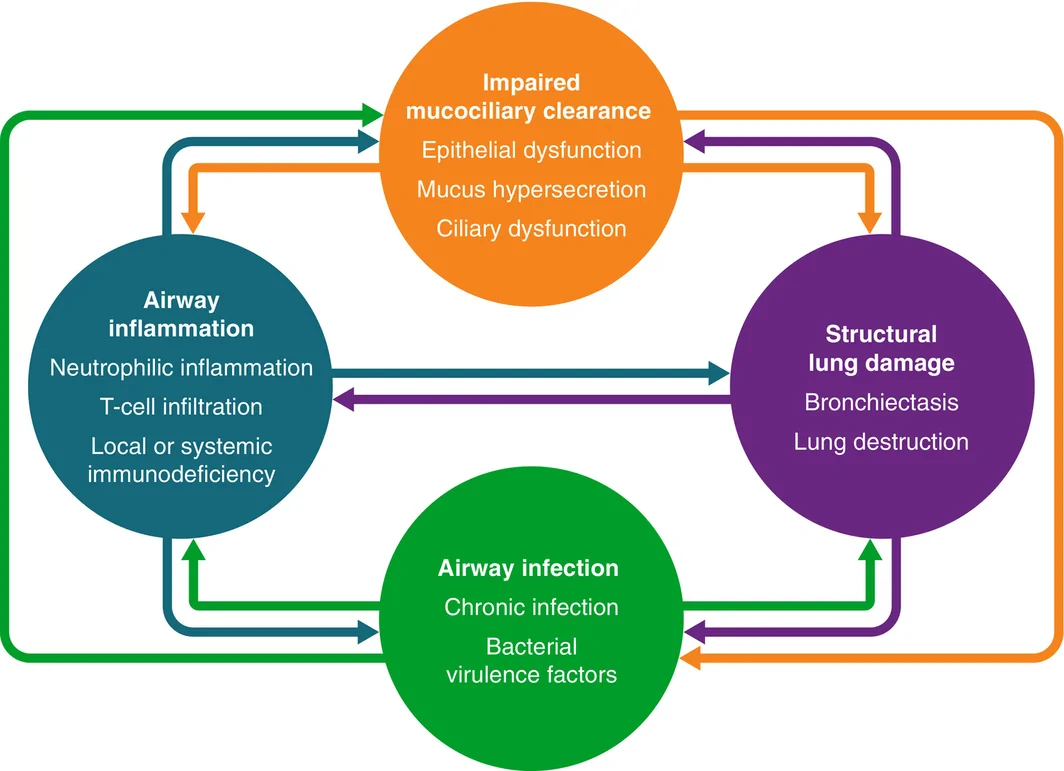
The vicious vortex of bronchiectasis. Adapted from Flume 2018 [4].

Bronchiectasis is a chronic, inflammatory lung condition characterized by permanently damaged and dilated bronchi, resulting in clinical symptoms such as chronic cough, sputum production, and frequent respiratory infections [1, 2]. Building on the vicious cycle theory of bronchiectasis originally proposed by Prof Peter Cole in 1986 [3], Flume and colleagues conceptualized the pathophysiology of bronchiectasis as a vicious vortex of four interrelated factors: airway inflammation, impaired mucociliary clearance, airway infection, and structural lung damage, as shown in Figure 1. Each component contributes to the initial development of bronchiectasis and drives subsequent disease progression [4].

Bronchiectasis typically manifests as periods of time where patients are relatively stable, interspersed with periods of acute clinical deterioration, or exacerbations. An exacerbation is defined as deterioration in three or more of the following key symptoms for at least 48 h: cough, sputum volume and/or consistency, sputum purulence, breathlessness and/or a decrease in exercise tolerance, fatigue and/or malaise, and haemoptysis, along with a clinician opinion that a change in management is required [5]. Exacerbations are associated with a decline in lung function, diminished quality of life, and increased morbidity and mortality [6, 7, 8]. Frequent exacerbations can accelerate the vicious vortex, leading to disease progression, and a history of exacerbations is a major predictor of future exacerbations [9]. The prevention and management of exacerbations of bronchiectasis is therefore a clinical priority.

The European Respiratory Society (ERS) published new guidelines for the management of bronchiectasis in September 2025 [8]. They provide evidence‐based recommendations for the care of people with clinically significant bronchiectasis, both when in the stable state and when experiencing acute exacerbations [8]. In this article, we retrospectively explore a complex case of bronchiectasis and discuss how the management of difficult exacerbations aligned with the new ERS guideline recommendations and where care could have been optimized.

## 2 Case History

**FIGURE 2:**
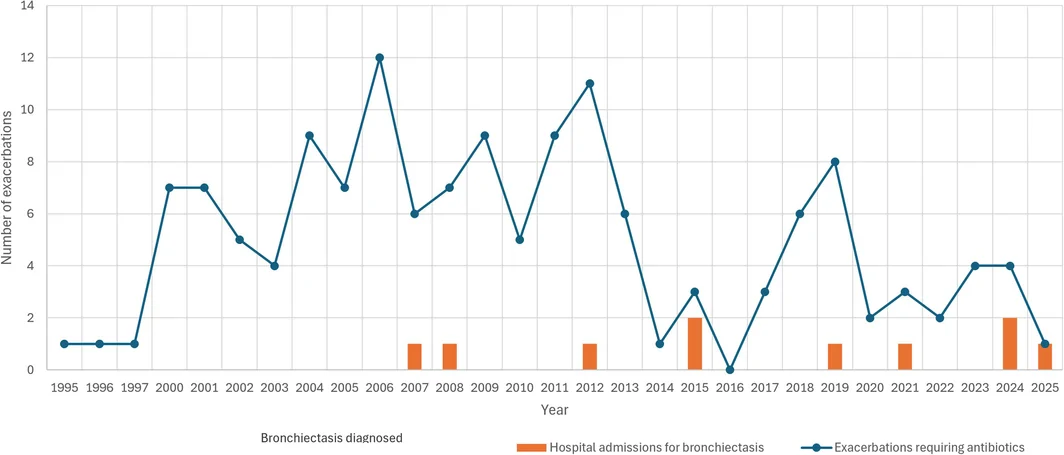
Number of exacerbations experienced by our patient per year. This article focuses on recent exacerbations of his bronchiectasis in the time period indicated by the red box.

Our case is a 75‐year‐old male who was diagnosed with bronchiectasis in 2005 at the age of 55 years. He has a prior history of asthma (diagnosed in 1991) and chronic obstructive pulmonary disease (COPD; diagnosed in 2004). These conditions are managed with inhaled corticosteroids (ICS), long‐acting β2‐agonists and long‐acting muscarinic antagonists. He is an ex‐smoker with 20 pack years who stopped smoking in 1990 at 40 years old. Serial CT scans show extensive bilateral bronchiectasis with mid and lower zone predominance. The patient has a Bronchiectasis Severity Index (BSI) score of 18 and is under the care of a specialist bronchiectasis hospital team in the UK. He experiences a large number of exacerbations per year as a result of all three of his chronic lung conditions, as shown in Figure 2.

We describe his case from July 2024, when he experienced a worsening of his symptoms including coughing up copious amounts of purulent sputum, breathlessness, and feelings of exhaustion. He recognized this as an exacerbation of his bronchiectasis and took a two‐week course of oral co‐amoxiclav, which he already had at home in a rescue pack provided by his GP. He did not improve, leading him to consult his GP, who prescribed another 2 weeks of co‐amoxiclav. However, his symptoms persisted, prompting him to contact his hospital team for advice.

### 2.1 Optimal Management of Acute Exacerbations

After contacting his hospital team, the patient was admitted to the respiratory virtual ward for treatment and monitoring, as his exacerbation had not resolved after 4 weeks of oral co‐amoxiclav treatment. This practice is consistent with the new ERS guidelines which recommend that patients who do not respond promptly to oral antibiotics should be reviewed to determine if there is a need for a change in treatment and/or hospitalization. Through the virtual ward, the patient could be monitored closely by the multidisciplinary team and provided with hospital‐level diagnostics and interventions, including the option to self‐administer treatments at home.

The new guidelines recommend that a sputum sample is obtained at the onset of an exacerbation before commencing antibiotic treatment. A sputum sample was indeed taken from the patient at this time, which grew *Proteus mirabilis* that was shown to be sensitive to co‐amoxiclav and co‐trimoxazole and *Candida* species. Due to the lack of response to oral antibiotics, a decision was made to commence intravenous (IV) antibiotics to treat the *P. mirabilis* infection. Despite this bacterium not being typically associated with bronchiectasis, it was thought to be the driver of the patient's exacerbation. Conversely, *Candida* species are common commensals in the respiratory tract and therefore do not usually require targeted therapy.

The patient was prescribed 1 g of IV meropenem to self‐administer at home three times a day for 2 weeks, which is consistent with the latest guidelines. There is very little evidence for the efficacy of home versus hospital IVs in patients with bronchiectasis, although one retrospective study carried out in adults [10] and one in children [11] suggests that home therapy results in similar clinical outcomes compared to inpatient therapy. Patients using home IVs may also benefit from a reduced risk of hospital‐acquired infections and an increase in quality of life compared with inpatient treatment. Surprisingly, the patient did not improve after 2 weeks of home IV treatment, which is recommended by the guidelines for treating exacerbations. He was switched to oral co‐trimoxazole, which he continued for 3 weeks in total.

We feel that the patient's lack of response to IV meropenem was likely related to a need to optimize airway clearance techniques (ACTs), rather than true antimicrobial resistance. A key part of bronchiectasis exacerbation management is the optimisation of ACTs, such as active cycle of breathing techniques and positive expiratory pressure (PEP) devices. These techniques target the impaired mucociliary clearance component of the viscous vortex and can improve sputum clearance, reduce the impact of cough, reduce the risk of exacerbations, and improve health‐related quality of life [12]. The latest guidelines recommend that ACTs may need to be adapted in frequency, intensity, and technique during an exacerbation. Consistent with the latest guidelines, our patient was referred to physiotherapy at this point to support sputum clearance during this exacerbation. However, due to long wait times, he did not see the respiratory physiotherapist until several months later. Resourcing issues limiting the provision of physiotherapy within bronchiectasis clinics are common but, given the importance of ACTs in managing the condition, it is imperative that organizations ensure prompt management is achievable in the future.

The patient's symptoms improved slightly in response to oral co‐trimoxazole, and he was discharged from the virtual ward. However, he was readmitted 2 weeks later as his sputum volume remained high, his cough was still bothersome, and he was still experiencing low energy levels. Consistent with the latest guidelines, another sputum culture was taken which showed heavy growth of *Pseudomonas aeruginosa*.

The guidelines recommend that eradication treatment is offered to patients with a new isolation of *P. aeruginosa*. This may refer either to the first time a patient has this bacterium isolated or a further isolation following a prolonged period during which *P. aeruginosa* was not detected. The guidelines acknowledge that eradication practices vary but state that regimens typically consist of 2 weeks of oral or IV antibiotics followed by 6–12 weeks of inhaled antibiotics. In line with this recommendation, the patient was rechallenged with 2 weeks of IV meropenem at home; however, he did not improve and so the course was extended by 2 weeks. He also visited the hospital daily to receive IV tobramycin 3 mg/kg once daily. The patient's serum tobramycin levels were monitored to ensure correct dosing and to check for renal toxicity. His renal function was determined to be stable and so the tobramycin dose was subsequently increased to 5 mg/kg once daily after 2 weeks due to poor response. His symptoms subsequently improved and he was discharged from the virtual ward. There is limited evidence for the use of dual antimicrobial approaches to treat *P. aeruginosa* in patients with bronchiectasis [13]. However, prior data in a cystic fibrosis population suggest that a combination of a beta‐lactam, such as meropenem, and an aminoglycoside, such as tobramycin, is beneficial for chronic infection [14]. Treatment decisions need to be balanced against the risk of nephrotoxicity and ototoxicity, particularly in older individuals.

Two months later, in December 2024, the patient contacted the virtual ward again to report feeling unwell with increased sputum production, breathlessness and feelings of tiredness, and exhaustion. He was re‐admitted to the virtual ward for another infectious exacerbation of his bronchiectasis. Consistent with the latest guidelines, two sputum samples were taken, which were both culture‐negative. However, he still felt unwell and further investigations revealed a raised C‐reactive protein level of 76 mg/dL, and an atypical infection was indicated on a CT scan. The patient was started on empirical oral co‐amoxiclav but did not show any improvement. A bronchoscopy was therefore performed and culture of the fluid showed moderate growth of *P. aeruginosa* resistant to tobramycin, colistin, and gentamycin, suggesting that the previous eradication attempt had failed. His antimicrobials were therefore switched to 2 g IV ceftazidime three times daily at home for 3 weeks.

At this point the patient was able to see the respiratory physiotherapist who taught him an ACT using a PEP device. However, he was unable to tolerate this as it exacerbated his cough and so he was given an oscillating positive expiratory pressure (OPEP) device instead. He was advised to do a slow expiratory phase in order to prevent coughing and to use his combination inhaler for COPD first to reduce broncho‐constriction and facilitate mucus clearance. A check of his technique confirmed that he was able to perform this accurately. He self‐reported improved ease of mucus clearance and decreased viscosity of mucus at his 6‐week physiotherapy follow‐up appointment.

The patient was now considered to have chronic infection with *P. aeruginosa*, and the latest guidelines strongly recommend that long‐term inhaled antibiotics are offered. Our patient had previously been on long‐term inhaled colomycin but lost response to this therapy and was switched to month‐on and month‐off tobramycin instead. Upon discharge from the virtual ward in March 2025, his nebulised tobramycin prophylaxis was escalated to continuous therapy to help control his exacerbations.

The latest guidelines also strongly recommend offering long‐term macrolides to patients at high risk of exacerbations despite standard care. The daily use of macrolide antibiotics, particularly azithromycin, has been shown to be an effective option for reducing exacerbation frequency in bronchiectasis patients, including those with chronic *P. aeruginosa* infection [8, 15, 16]. Consistent with the latest guidelines, our patient started on 500 mg prophylactic azithromycin three times a week in early 2025, alongside his nebulised tobramycin. In line with the ERS guidelines and British Thoracic Society (BTS) long‐term macrolide guidelines [17], he was screened for nontuberculous mycobacterial (NTM) infection, QTc prolongation, and liver/kidney function abnormalities prior to starting therapy, and this was checked again 4 weeks after initiation. He is tolerating this prophylaxis therapy well and remains stable.

### 2.2 Ongoing Bronchiectasis Management to Reduce Frequency and Severity of Exacerbations

As well as promptly treating exacerbations as they occur, it is also important to take a holistic approach to bronchiectasis management in order to optimize outcomes over the long‐term. The latest guidelines recommend that mucoactive treatments are offered to patients with bronchiectasis where airway clearance has failed to control symptoms. These treatments include drugs such as hypertonic saline and carbocisteine, which are used to help patients expectorate mucus using ACTs [18]. The guidelines acknowledge the limited evidence around their efficacy and a recent study has shown that neither hypertonic saline nor carbocisteine significantly reduced the mean incidence of pulmonary exacerbations in patients with bronchiectasis [19]. Further studies are required to better understand the role of mucolytics in this patient population. Our patient used nebulised sodium chloride 0.9% from 2008 to 2023, although there is no evidence for the use of this formulation and it perhaps reflects out‐of‐date practice. From 2020 to 2021, he used nebulised hypertonic saline 7% twice a day with very little effect aside from slightly thinning his sputum. Unfortunately, the physiotherapy treatment burden led to the patient declining to restart hypertonic saline during his last exacerbation in early 2025. He also takes 750 mg carbocisteine three times a day with symptomatic improvement and no reports of side effects.

Pulmonary rehabilitation (PR) is an exercise and education program designed for people with lung disease who experience symptoms of breathlessness [20]. The latest guidelines recommend that patients with breathlessness and/or impaired exercise capacity should be offered PR. However, many PR programs in the UK are primarily targeted at patients with COPD. Our patient attended a PR course in 2014 to help him manage his COPD, but the outcomes of this are unknown.

The latest guidelines suggest not to offer long‐term inhaled corticosteroids to patients with bronchiectasis due to a lack of evidence of efficacy and risk of infections, such as NTM and pneumonia. However, our patient uses them as long‐term maintenance treatments for his asthma and COPD, and the guidelines recognize that the presence of bronchiectasis should not alter the recommendation to use ICS in such patients. The patient is seen at his GP surgery for annual asthma and COPD reviews, where his inhaler technique is checked to ensure that the appropriate device is being prescribed and that the lowest effective dose is being used to minimize the risk of adverse effects.

The latest guidelines recommend that bronchiectasis patients remain up to date with key vaccinations for respiratory pathogens. Consistent with this, our patient receives the influenza and COVID‐19 vaccine every year and is up to date with vaccinations for *Herpes zoster*, respiratory syncytial virus, pneumococcal disease, and swine influenza (H1N1).

### 2.3 Outcome and Follow‐Up

Our patient has remained stable since March 2025 and has not required any antibiotics for infectious exacerbations of his bronchiectasis. His last sputum sample, sent in April 2025, was culture‐negative. The patient is reviewed by a respiratory consultant every 3–4 months to assess his response to inhaled antibiotics and to determine whether a change in treatment is required. If he experiences another worsening of his bronchiectasis symptoms, there is a plan in place for him to contact the virtual ward again for advice.

## 3 Discussion

The ERS guidelines recommend that patients who experience two or more exacerbations per year or who have had one severe exacerbation in the previous year should be considered for more frequent follow‐up and a lower threshold for treatment [8]. Our patient falls into this category, with a BSI score of 18. The retrospective review of this case highlights many areas where optimal practice was achieved consistent with the recently published ERS guidelines, such as prompt sputum cultures at the start of an exacerbation, the rapid initiation of antibiotics, and regular reviews of treatment efficacy. It also identified areas for enhancing future care for patients, which could include more timely access to respiratory physiotherapy in an outpatient setting and re‐referral to PR.

Our patient has a self‐management plan that includes guidance on recognizing exacerbations, as recommended by the latest guidelines. This enabled him to appropriately self‐administer oral antibiotics from a rescue pack at home 48 h after his respiratory symptoms worsened. The ability to start treatment at this early stage is vital to prevent disease progression, loss of lung function, and reductions in quality of life.

Access to a virtual ward has been particularly important for our patient, as he was able to promptly and easily obtain support from a specialist respiratory team for his exacerbations. In 2023, the NHS launched a national programme, aiming to increase the provision of hospital‐level care to patients at home [21]. This allows close observation of symptoms, rapid intervention if a patient deteriorates, and earlier supported discharge whilst reducing pressure on the healthcare system [22, 23]. In our experience, patients generally prefer admission to the virtual ward instead of the hospital, as they feel more comfortable at home and recover more quickly in this familiar environment.

The complex nature of our patient's bronchiectasis means that he has been prescribed many different antibiotics to control each of his exacerbations. However, each prescription was guided by microbiology results, the patient's response to treatment and his tolerance of each therapy, which is consistent with recommendations in the latest guidelines. He also takes long‐term antibiotics to help prevent future exacerbations, for which antimicrobial stewardship is a key consideration. The guidelines state that long‐term antibiotic treatment should be used only after other aspects of management have been optimized, including ACTs, vaccination against respiratory pathogens, treatment of the underlying cause of bronchiectasis, and assessment of co‐morbidities [8]. Our patient has a chronic *P. aeruginosa* infection, is practicing airway clearance and is up to date with vaccinations, justifying the use of long‐term antibiotics in conjunction with confirmation of response based on regular reviews. Another key issue to consider when prescribing inhaled antibiotics, such as nebulised tobramycin, is the additional burden that they place on patients due to the time required for their administration and subsequent cleaning of equipment.

Our patient had a lengthy wait to see a respiratory physiotherapist, sadly reflecting real‐life disparities in access across the UK. Respiratory physiotherapy is an essential component of bronchiectasis management but is often under‐prioritized by patients. When prescribing ACTs and mucolytic agents, it is important to personalize treatment to balance efficiency and burden to the patient, especially as there is no evidence for the superiority of one ACT or mucolytic over another [12, 19]. The factors influencing personalization are varied and include the patient's physical ability, patient preference, the intervention type, and the frequency and duration of the intervention [24]. Another method to support personalization of ACTs could involve shared decision‐making. This is an approach where clinicians and patients work together to choose an ACT the patient may prefer, which could support increased adherence to this key therapy [25].

We welcome publication of the latest ERS guidelines, which may help NHS organizations benchmark and enhance their bronchiectasis services going forward to optimize patient care. There is also an opportunity for the guidelines to be used to support local business cases to improve access to essential physiotherapy services.

## Author Contributions

**Hulance Bianca:** conceptualization, writing – original draft, methodology, visualization, writing – review and editing, data curation, validation, resources, investigation. **Capstick Toby:** conceptualization, writing – original draft, methodology, validation, visualization, writing – review and editing, data curation, resources, investigation. **Shah Anand:** conceptualization, writing – original draft, methodology, validation, visualization, writing – review and editing, data curation, resources, investigation. **McCallion Paul:** conceptualization, writing – original draft, methodology, validation, visualization, writing – review and editing, data curation, resources, investigation.

## Funding

Insmed Limited provided funding to Axiom Health Ltd. for medical writing and editorial support.

## Ethics Statement

The authors have nothing to report.

## Consent

The authors have obtained written informed consent from the patient.

## Conflicts of Interest

All authors report medical writing and editorial support paid to Axiom Health Ltd. by Insmed for the present manuscript. All authors report honoraria for an initial advisory board meeting to discuss the present manuscript from Insmed. BH reports support for attending meetings and honoraria for participation in an advisory board from Insmed. TC reports a research grant from Chiesi, consulting fees from AstraZeneca, GSK, and Sanofi, professional speaker fees from AstraZeneca, Chiesi, GSK, Insmed, and Orion, and support for attending meetings from AstraZeneca, Chiesi and GSK. In addition, TC reports holding the following positions: Committee Member of the UK Clinical Pharmacy Association—Respiratory Group, Clinical Service Adviser and Steering Group Member of the UK MDR‐TB Clinical Advice Service, Director of The UK Inhaler Group and Management Group Member of NTM Network UK. AS reports research grants from AstraZeneca and Argenx, consulting fees from Argenx, Mundipharma, and Pfizer, professional speaker fees from Pfizer, and participation in an advisory board for Kymera. PM reports a doctoral fellowship grant from the National Institute for Health and Care Research, professional speaker fees from ICU Medical, Inogen, and Insmed, and participation in an advisory board for Insmed.

## Data Availability

Data sharing is not applicable to this article as no datasets were generated or analyzed.
